# The importance of considering regulatory domains in genome-wide analyses – the nearest gene is often wrong!

**DOI:** 10.1242/bio.059091

**Published:** 2022-04-04

**Authors:** Ellora Hui Zhen Chua, Samen Yasar, Nathan Harmston

**Affiliations:** 1 Science Division, Yale-NUS College, Singapore 138527, Singapore; 2 Program in Cancer and Stem Cell Biology, Duke-NUS Medical School, Singapore 169857, Singapore

**Keywords:** Chromatin structure, Enhancers, Gene regulation

## Abstract

The expression of a large number of genes is regulated by regulatory elements that are located far away from their promoters. Identifying which gene is the target of a specific regulatory element or is affected by a non-coding mutation is often accomplished by assigning these regions to the nearest gene in the genome. However, this heuristic ignores key features of genome organisation and gene regulation; in that the genome is partitioned into regulatory domains, which at some loci directly coincide with the span of topologically associated domains (TADs), and that genes are regulated by enhancers located throughout these regions, even across intervening genes. In this review, we examine the results from genome-wide studies using chromosome conformation capture technologies and from those dissecting individual gene regulatory domains, to highlight that the phenomenon of enhancer skipping is pervasive and affects multiple types of genes. We discuss how simply assigning a genomic region of interest to its nearest gene is problematic and often leads to incorrect predictions and highlight that where possible information on both the conservation and topological organisation of the genome should be used to generate better hypotheses.

The article has an associated Future Leader to Watch interview.

## Abstract (precis)

**Summary:** Identifying which gene is the target of an enhancer is often accomplished by assigning it to the nearest gene, here we discuss how this heuristic can lead to incorrect predictions.

## Introduction

The precise regulation of gene expression is necessary for both development and homeostasis, with dysregulation leading to developmental disorders and disease (Lee and Young, 2013). In multicellular organisms, a number of genes are not primarily regulated by their core and proximal promoter, but are under the control of regulatory elements located both proximally and distally, often referred to as enhancers (Miguel-Escalada et al., 2015). Chromatin looping between enhancers and promoters places these elements into close physical proximity with their cognate target genes, with this spatial colocalisation being necessary for their role in regulating gene expression (Tolhuis et al., 2002). While genes and their regulatory elements are organised on a linear chromosome, within the nucleus they are part of a complex, hierarchical and non-random three-dimensional structure (Lieberman-Aiden et al., 2009). The development of experimental techniques to investigate this topological organisation has provided insights into how the spatial conformation of chromatin across multiple levels directly affects the regulation of gene expression.

Only ∼2% of the human genome is involved in coding for proteins, while at least 8.2% is under some level of selective pressure (Rands et al., 2014), indicating the importance of non-coding regions. Depending on the transcription factor (TF) or histone modification investigated, a large proportion of ChIP-seq peaks are located outside of coding regions (i.e. are intergenic or intronic), and 93% of single nucleotide polymorphisms (SNPs) identified by genome-wide association studies (GWAS) have been found to be located in non-coding regions (Maurano et al., 2012). Maurano et al. reported that 76.6% of GWAS SNPs lie within a regulatory region defined by DNase I hypersensitivity or were in linkage disequilibrium (LD) with SNPs overlapping a DNase hypersensitive site (DHS) (Maurano et al., 2012).

Enhancers drive expression in a cell-type specific and spatiotemporal manner, potentially regulating only one specific gene in one specific context (Long et al., 2016). These elements are bound by TFs which provide regulatory logic (Jindal and Farley, 2021), and are associated with distinct patterns of histone modifications (Creyghton et al., 2010). Unlike SNPs in protein coding genes, which may affect splicing or protein structure/function, SNPs in regulatory elements can result in changes in expression of the gene they are regulating. SNPs frequently alter allelic chromatin state and disrupt TF binding sites (TFBSs), directly implicating non-coding variation in regulatory elements as drivers of both phenotypic diversity and disease (Kasowski et al., 2010; Spielmann and Mundlos, 2016). The regulatory domain, or regulatory landscape (Acemel et al., 2017), of a gene is understood to be the region of the genome which contains all of the regulatory information and elements which allows the gene to be expressed correctly (Bolt and Duboule, 2020). With enhancers responsible for regulating the expression of one or several genes, in different contexts, being located throughout this region.

One commonly used method for assigning a non-coding locus of interest (i.e. enhancer or SNP) to its target/relevant gene is to assign it to the nearest gene on the physical chromosome. Here, we discuss the existing experimental evidence, at individual loci and genome-wide, and using insights from comparative genomics, that nearest gene assignment is often misleading, and highlight the importance of considering the topological architecture of the genome when annotating and interpreting the results from genome-wide experiments.

**Table 1.**
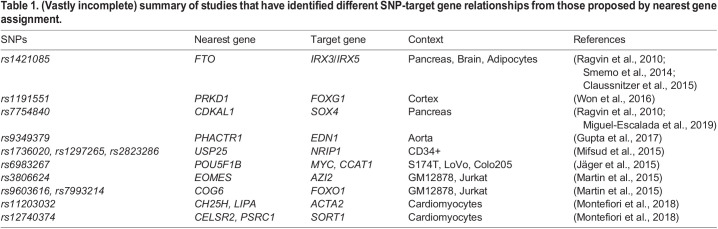
**(Vastly incomplete) summary of studies that have identified different SNP-target gene relationships from those proposed by nearest gene assignment.**

## The topological organisation of the genome determines the range of regulatory interactions

**Fig. 1.**
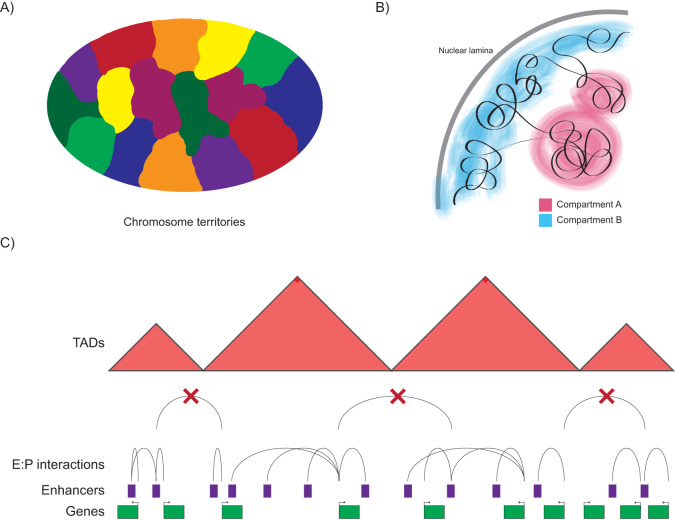
**Chromatin within a nucleus is hierarchically organised.** (A) Chromosomes occupy distinct regions or territories within the nucleus known as chromosome territories. (B) Chromosomes are partitioned into compartments, with compartment A being associated with the interior of the nucleus and active chromatin, compartment B being associated with inactive chromatin located near the nuclear lamina. (C) The genome is further partitioned into TADs, with interactions between enhancers and their target genes preferentially occurring within TADs. At several TADs, enhancers regulating the expression of a gene can be located throughout its TAD – indicating that TADs can directly correspond to gene regulatory domains.

Within the nucleus, chromosomes are located in spatially distinct chromosome territories (Fig. 1A) (Bolzer et al., 2005; Cremer and Cremer, 2010). Chromatin is organised into active (A) and inactive (B) compartments (Fig. 1B), with the genome further partitioned into a set of preferentially interacting regions, known as topologically associating domains (TADs) (Shopland et al., 2006; Lieberman-Aiden et al., 2009; Dixon et al., 2012, 2015) (Fig. 1C). The partitioning of the genome into compartments occurs because of preferential interactions between regions of chromatin which have a similar state, with compartment B being associated with inactive, gene-poor regions of the genome, which are preferentially found in close proximity to the nuclear lamina, whereas compartment A is associated with active, gene-rich regions of the genome that are often located towards the centre of the nucleus and are often associated with transcription factories. During differentiation, chromatin can switch compartment type, from A to B or vice versa, and this is associated with changes in expression of the genes in this region (Dixon et al., 2015). Genes and regulatory elements located within a TAD preferentially interact with each other but show a depletion for interactions with chromatin located in adjacent TADs, suggesting that TAD boundary regions act as insulators and that they function to constrain the range and activity of regulatory elements (Dixon et al., 2012).

TADs have been found to represent coherent functional blocks in terms of their association with replication domains, patterns of CTCF conservation and lamina associated domains (Paulsen et al., 2017; Pope et al., 2014; Vietri Rudan et al., 2015), and are largely invariant across cell types (Siersbæk et al., 2017). Several studies support the direct concordance between TADs and gene regulatory domains. Genome-wide studies of enhancer–promoter interactions have found that the clear majority of interactions occur within a TAD (Montefiori et al., 2018; Jung et al., 2019). Random insertion of enhancer reporter constructs into the mouse genome found that the patterns of reporter activation during development reflected the organisation of a subset of TADs and the expression of their constituent genes (Symmons et al., 2014). At several loci, perturbation of TAD boundaries, via deletion of CTCF boundary sites and other techniques, has been demonstrated to lead to ectopic enhancer–promoter interactions and dysregulation of gene expression (Lupiáñez et al., 2015; Franke et al., 2016; Narendra et al., 2016; de Bruijn et al., 2020). However, other studies have found that perturbing TAD structure and boundaries does not have a large effect on gene expression (Despang et al., 2019; Ghavi-Helm et al., 2019). Mutations affecting TAD boundaries have been associated with ectopic enhancer–promoter interactions and altered gene expression in a number of neurological and developmental disorders (Lupiáñez et al., 2015; Franke et al., 2016). In tumors, structural variation affecting TAD organisation has been identified in multiple types of cancer (Taberlay et al., 2016; Akdemir et al., 2020), with experimental evidence that the deletion or rearrangement of boundary regions can directly lead to the dysregulation of important oncogenes by allowing ectopic enhancer–promoter interactions (Vicente-García et al., 2017; Weischenfeldt et al., 2017).

Therefore, at multiple loci, TADs appear to function as structural units of the genome whose purpose is to increase the probability that regulatory elements meet their target promoters within a specific domain, whilst decreasing the probability of interacting with elements and genes outside of the domain (Flyamer et al., 2017), helping to ensure that genes are turned on and off by the correct enhancers. However, it is likely that TADs may have multiple other functions (Austenaa et al., 2015; Hu et al., 2015), and that the correspondence between TADs and regulatory domains is likely only seen at a distinct subset of loci.

## Patterns of microsynteny in metazoan genomes as a consequence of gene-regulatory constraints

During evolution, genomes can be rearranged leading to differences in the order and location of genes between species. However, the observed patterns of gene order (microsynteny) are not random (Oliver and Misteli, 2005), with hundreds of genes being found to be physically linked together over large evolutionary distances (Irimia et al., 2012). This has been proposed to be the result of either the co-expression/co-regulation of these genes, or that one member of the pair (the *bystander gene*) contains regulatory elements within its introns or exons that are necessary for the proper regulation of the other (the *target gene*).

This pattern of microsyntenic conservation, reflecting the need to keep regulatory elements in *cis* with their target genes was found to overlap with loci that have a high density of conserved non-coding elements (CNEs) (Bejerano, 2004). CNEs have a high percentage identity over a large number of base pairs between evolutionarily distinct groups of species, and often display regulatory activity in reporter assays (Harmston et al., 2013). Several of these CNEs were found to be located within the introns of housekeeping genes but were involved in the regulation of a different gene(s). The combination of these features led to the proposal of the genomic regulatory block (GRB) model (Becker and Lenhard, 2007; Engström et al., 2007; Kikuta et al., 2007b), where genes are physically linked together because of the need to ensure proper developmental regulation of a specific target gene(s). This has led to the linkage of developmental transcription factors with housekeeping genes over large evolutionary distances, with these linkages spanning large genomic distances. Investigation of these regions using retroviral screens identified that insertions of reporter genes around important transcription factors resulted in the same expression pattern regardless of their position, highlighting that these regions correspond to the regulatory domains of these important transcriptional regulators (Kikuta et al., 2007a; Navratilova et al., 2009).

Studies have found that TADs are syntenic between humans and mice (Dixon et al., 2012) and show conservation in macaques and dogs (Vietri Rudan et al., 2015), with chromosomal rearrangements preferentially occurring at the boundaries of TADs (Berthelot et al., 2015; Liao et al., 2021). A subset of TADs directly corresponds to the location of GRBs, as inferred from the distribution of CNEs, across multiple species (Harmston et al., 2017). The correspondence between GRBs and TADs suggests that this subset of TADs primarily corresponds to the regulatory domain of a developmental TF under long-range regulation and not all of its constituent genes. Recent studies directly comparing TAD organisation in *Drosophila melanogaster* and *Drosophila triauraria* identified conservation of a subset of TADs with distinct features, further supporting this observation (Torosin et al., 2020). Conservation of TADs and maintenance of microsynteny both reflect selective pressure on genome organisation due to gene regulatory constraints, further supporting the notion that TADs reflect ‘regulatory units’ of the genome (Dixon et al., 2016) and gene-regulatory domains.

The identification that elements responsible for regulating the expression of one gene can be located within other genes directly highlights a problem with nearest gene assignment. These enhancers would be annotated as regulating their overlapping gene, which at many loci throughout the genome would be incorrect.

## Studies of individual loci illuminate the fallacy of nearest gene assignment

**Fig. 2.**
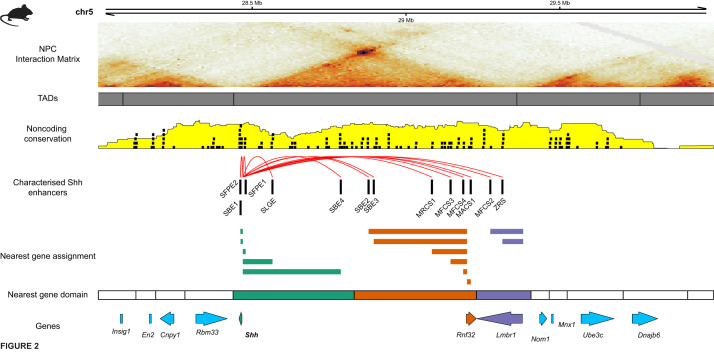
**The regulatory domain of the key developmental transcription factor *Shh*.** Visualisation of the region chr5:28Mb-30Mb in mouse (mm10) which contains multiple genes; displaying the Hi-C interaction matrix in neural progenitor cells (NPC), TADs and locations of validated *Shh* enhancers. *Shh* is under the control of multiple enhancers located throughout its TAD, some of which are located closer to other genes than *Shh*, which would be erroneously assigned by using nearest gene assignment.

Sonic Hedgehog (*SHH*) is a key developmental transcription factor with important roles in development of several tissues (Briscoe and Thérond, 2013). In mammals, *SHH* lies within a large TAD spanning approximately 920 kb (Fig. 2) with a high density of CNEs. *SHH* is under complex enhancer-driven regulation by multiple elements located both proximally and distally (Anderson et al., 2014). Several enhancers have been identified within this TAD that drive *SHH* expression in a variety of tissues, including the brain, laryngotracheal tube, gut and limb bud (Lettice, 2003; Jeong et al., 2008; Sagai et al., 2009; Tsukiji et al., 2014) (Fig. 2). Several mutations within the regulatory domain surrounding *SHH* have been found to cause congenital abnormalities (Hill and Lettice, 2013). Genetic mapping of an interval associated with preaxial polydactyly (Hing et al., 1995) implicated *LMBR1* as a putative regulator of limb development (Clark et al., 2000). However, further studies found that proper expression of *SHH* in the developing limb bud depends on an enhancer (known as ZRS) located within the fifth intron of *LMBR1*, located 850 kb distally from *SHH* (Lettice et al., 2002; Lettice, 2003). This intronic enhancer corresponds to a CNE which has identifiable sequence conservation back to shark (Dahn et al., 2007); with this region of the genome being classified as a GRB. Polymorphisms within this element result in limb defects, including preaxial polydactyly and syndactyly in human (Lettice et al., 2008), with deletion of this element leading to limb truncation in the mouse (Sagai et al., 2005). A number of these mutations are responsible for altering the activity and specificity of TFBSs located within ZRS, perturbing both the level and extent of *SHH* expression in the developing limb bud (Zhao et al., 2009; Lettice et al., 2012). Insufficient expression of *SHH* during brain development results in holoprosencephaly, which can be caused by mutations either affecting the coding region of *SHH* (Roessler et al., 1996) or its regulatory landscape (Belloni et al., 1996). Translocation events in the vicinity of *SHH* have been found to displace enhancers away from the *SHH* promoter (Jeong et al., 2006), and mutations within SBE2 have been shown to directly affect the expression of *SHH* by altering *Six3* binding at this enhancer (Jeong et al., 2008). Investigation of the *Shh* regulatory domain by transposon insertion mapping revealed that the range of action for *Shh* enhancers is coherent with the span of the regulatory domain predicted using Hi-C, and that the neighbours of *Shh* did not respond to long-range regulation (i.e. *Rnf32*) (Anderson et al., 2014). Considering the genomic locations of functionally characterised enhancers of *Shh* (*N*=13, Fig. 2), eight (61%) would be associated with the wrong gene by nearest gene assignment, including ZRS and SBE2.

**Fig. 3.**
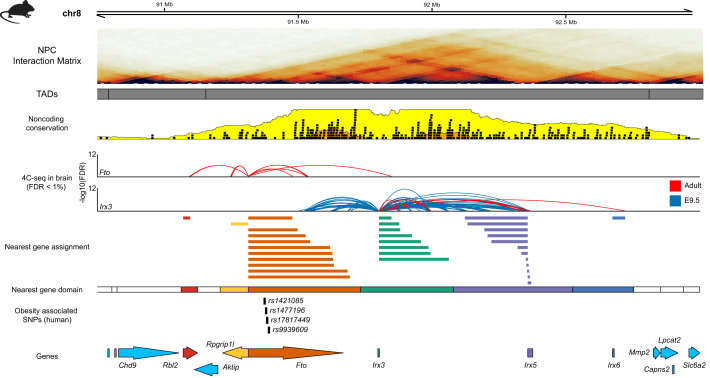
**The regulatory domain of the key developmental transcription factors Irx3/5 and 6.** Visualisation of the region chr8:90.5Mb-93Mb in mouse (mm10); displaying Hi-C interaction matrix from in neural progenitor cells (NPC), location of interactions as identified 4C involving the promoters of *Fto* and *Irx3* (Smemo et al., 2014)*. Irx3* is regulated by multiple regulatory elements located throughout its TAD, including elements located within the introns of *Fto*.

Variants located within *FTO* (Gerken et al., 2007), fat-mass and obesity-associated gene, have been associated with several obesity-related phenotypes using GWAS (Dina et al., 2007; Frayling et al., 2007). The set of variants identified at this locus have been found to be highly replicable, have a high population frequency and show a strong effect size (Scuteri et al., 2007). Although these SNPs are located within introns 1 and 2 of *FTO*, eQTL studies found no evidence of a link between them and differences in the expression and splicing of *FTO* (Grunnet et al., 2009; Klöting et al., 2008). *FTO* is located within a region enriched for extreme non-coding conservation identified as a GRB (de la Calle-Mustienes et al., 2005), which accurately predicts the boundaries of the topological domain at this locus (Harmston et al., 2017; Hunt et al., 2015). The predictions of the GRB model indicate that the majority of regulatory elements within this region are directly involved in the regulation of *IRX3* and *IRX5* (the target genes of this GRB), and that *FTO* and *RPGRIP1L* are simply bystander genes that are only regulated by proximal regulatory elements, if at all. This prediction has been confirmed by results from a number of studies. Enhancer screens have demonstrated that the CNE containing *rs1421085,* a SNP associated with Type 2 diabetes (T2D)*,* acts as an enhancer driving reporter expression in the pancreatic area at 48 hpf in zebrafish (Ragvin et al., 2010). This suggested that the GWAS signal was not reflecting a variant associated with regulation of the constitutively expressed *FTO,* but an enhancer variant affecting the expression of *IRX3* and *IRX5*. Smemo et al. demonstrated that *FTO* is not under long range regulation in the mouse brain, but that *IRX3* interacts with intronic elements located within *FTO* (Smemo et al., 2014) (Fig. 3). In addition, studies have found that the *FTO* promoter does not respond to long-range regulation during zebrafish development (Rinkwitz et al., 2015). Recently, it has been shown that *rs1421085* is located within an *ARID5B* binding site, leading to impaired *ARID5B*-mediated repression of *IRX3* and *IRX5* during early adipocyte differentiation (Claussnitzer et al., 2015). This loss of repression leads to a loss of mitochondrial thermogenesis and a shift from fat browning to whitening programs. Therefore, although these variants appeared to implicate *FTO* as the causative gene based on nearest gene assignment, multiple experiments across several species and tissues have provided extensive evidence, and importantly mechanistic explanations, for these variants being involved in affecting the enhancer-driven regulation of *IRX3* and *IRX5*.

Additional studies have also shown the pervasiveness of genes involved in interactions with elements at ranges beyond the nearest gene at a multitude of other loci. *MEIS1* is located within a region containing a large number of CNEs. Several of these CNEs were tested in enhancer assays, with 65% (22/34) testing positive and recapitulating the expression patterns of *MEIS1* (Royo et al., 2012). Using the heuristic of nearest gene assignment, eight (36%) of these elements would be assigned to different protein-coding genes. In zebrafish, regulatory elements located within the introns of the skin-specific *slc2a15a* and ubiquitously expressed *fbxw4* are involved in the regulation of *Fgf8a* (Komisarczuk et al., 2009). This study found that the majority of regulatory elements within this region drove expression of *Fgf8a*, with *slc2a15a* and *fbxw4* appearing to be non-responsive to long-range regulation. The ability of elements located within *fbw4* to drive *Fgf8* expression has been confirmed in mice (Marinić et al., 2013). In addition, the loss of exonic enhancers located within *DYNC1I1* in humans has been reported to lead to split hand/foot malformations by affecting the expression of *DLX5/6, a* gene located 1Mb from these enhancers (Lango Allen et al., 2014). Therefore, multiple studies investigating regulatory elements at single loci have found that initially annotating the nearest gene as the target of an enhancer and/or genetic variant is often incorrect.

## Insights from integrative genome-wide analyses of topological organisation

Although techniques including 3C and 4C permit the assessment of interactions between pre-determined viewpoints and one or multiple genomic regions, a number of techniques have enabled the identification of chromatin interactions genome-wide (McCord et al., 2020). Analyses based on Hi-C data have helped to putatively define the *cis*-regulatory domains and have identified mechanisms and factors involved in regulating chromatin looping (Phillips-Cremins et al., 2013; Van Bortle et al., 2014). However, significantly higher resolution data is required to precisely identify interactions between regulatory elements and promoters, which can be achieved by including an enrichment step (Fullwood et al., 2009; Schoenfelder et al., 2015; Mumbach et al., 2016), using a different restriction enzyme (i.e. that cuts DNA more frequently) and/or by sequencing to a higher depth (Rao et al., 2014; Hua et al., 2021). Studies of chromatin interactions using these techniques, and integration of these maps with other types of data, have identified a number of important features relevant to understanding nuclear organisation and gene regulation, and have proposed new genes as being involved in disease processes.

Insertional mutagenesis screens use the patterns of recurrent retrovirus insertions, known as common insertion sites, to identify genes potentially involved in tumourigenesis (Uren et al., 2008). These insertions can lead to changes in gene expression with subsequent effects on tumour growth. However, insertions may be located anywhere within the regulatory domain of the gene whose expression it disrupts. By considering retroviral insertions in the context of spatial organisation, as defined using Hi-C, Babaei et al. were able to identify novel target genes that were not originally proposed using nearest gene assignment and re-assigned some insertions as putatively regulating different genes (Babaei et al., 2015). Several of these genes (i.e. *BRCA2*, *FANCS*, *APC*, *JAK1*, *NOTCH1*) were proposed to be more probable targets than the originally reported genes that were located near to insertions, with insertions involved in interactions being found to be more likely to deregulate genes involved in tumourigenesis. Therefore, integrating information on topological organisation with patterns of retroviral insertions led to improvements in sensitivity and specificity, and subsequently to new biological hypotheses.

Integrating Hi-C maps of the developing brain with the results from schizophrenia GWAS (Schizophrenia Working Group of the Psychiatric Genomics Consortium, 2014) identified 402 genes that were involved in interactions with a region containing a significantly associated SNP, but which were neither adjacent to, nor in LD with this SNP (Won et al., 2016). This set of genes was enriched for processes including neuronal differentiation, and significantly overlapped with a set of genes known to be downregulated in the prefrontal cortex of schizophrenia patients. *rs1191551,* an SNP associated with schizophrenia, was identified as being located within an enhancer active in the developing cortex, which was interacting with *FOXG1* (located 760 kb away) instead of the nearby *PRKD1* (located 45 kb away). Deletion of this region led to a decrease in expression of *FOXG1*, but had no effect on *PRKD1*, confirming that this region is involved in regulating *FOXG1*.

Capture Hi-C (cHi-C) combines Hi-C with target sequence enrichment (i.e. using baits targeting specific restriction fragments of interest) to improve resolution without requiring the sequencing of exponentially more reads (Schoenfelder et al., 2015). This technique allows the investigation of the interactions that a set of pre-defined genomic features (i.e. promoters or GWAS SNPs) are involved in at lower cost than standard Hi-C, essentially trading library complexity for statistical power. Mifsud et al. performed cHi-C in human blood cell lines and calculated that two thirds of promoter-centred interactions appeared to interact with the nearest gene, with the remainder interacting over longer distances, often across intervening genes (Mifsud et al., 2015). *USP25* is the closest gene (∼280 kb away) to three SNPs implicated in inflammatory bowel disease, but all of these SNPs were observed to interact with *NRIP1*, a gene located ∼380 kb away.

Targeting loci associated with susceptibility to autoimmune disease in B- and T-cells identified cell-type specific interactions between elements located near disease-associated SNPs and genes which were not adjacent in the genome (Martin et al., 2015). The 3′ intronic region of *COG6* contains a number of SNPs associated with rheumatoid arthritis (RA) and juvenile idiopathic arthritis (JIA) which exhibited robust interactions with *FOXO1*, located over 1 Mb away. RA-associated variants located close to *EOMES* were identified as being involved in long-range interactions, spanning approximately 640 kb, with the promoter of *AZI2*. *DEXI* interacted with multiple loci associated with susceptibility to different autoimmune diseases over long distances; interacting with a region associated with type 1 diabetes and JIA located adjacent to *RMI2* (∼530 kb away) and an RA-associated locus proximal to *ZC3H7A* (∼1.2 Mb away). Three distinct SNPs located at 6q23 have been independently associated with various autoimmune diseases. A study investigating this locus using cHi-C revealed that these SNPs were involved in interactions with multiple genes over a wide range of distances and intervening genes (Martin et al., 2016). Although extensive work has suggested an important role for *TNFAIP3* in the modulation of autoimmune disease, the use of conformation capture techniques implicated additional putative genes and suggested that the primary causal gene may be *IL20RA* (Martin et al., 2016). Both of these studies demonstrate that non-coding variants associated with similar diseases may be involved in regulating the same gene, or genes in the same pathway, despite them being located distally from each other and proximal to other genes.

Promoter capture Hi-C (pcHi-C) maps of iPSCs and iPSC-derived cardiomyocytes found that 90% of interactions involving SNPs associated with cardiovascular disease and their target genes did not involve the nearest gene, with the majority (89%) of these interactions being between genes and regulatory elements located within the same TAD (Montefiori et al., 2018). Several SNPs associated with cardiovascular disease are proximal to both *CELSR2* and *PSRC1*, however the region containing these SNPs was found to interact with *SORT1* located 120 kb away, supporting a previous study that *SORT1* is the target gene of this region (Musunuru et al., 2010). While *rs11203032* is proximal (<10 kb) to both *CH25H* and *LIPA,* this region was found to be interacting with *ACTA2*, located 220 kb away, indicating that it is actually the correct target gene.

By performing pcHi-C in human pancreatic islets, Escalada et al. were able to propose target genes for regulatory elements overlapping with type 2 diabetes (T2D) and fasting glycemia SNPs (Miguel-Escalada et al., 2019). This identified potential target genes for 53 regions, however only 24 of these regions (45%) interacted with the gene annotated in the original GWAS. 87% of these regions were found to interact with at least one gene not previously annotated by GWAS, including the regulation of *SOX4* by intronic *CDKAL1* enhancers, as previously proposed (Ragvin et al., 2010). Several of these putative SNP:gene interactions were validated experimentally using CRISPR. *rs11257655* was proximal to *CDC123* (∼15 kb), however this region was found to be interacting with more distally located genes, *OPTN* and *CAMK1D* (located 834 kb and 84 kb away respectively). CRISPR deletion of the enhancer overlapping *rs11257655* led to downregulation of both *CAMK1D* and *OPTN*, with no effect on *CDC123*. In all of the loci tested, deletion of the regulatory element affected the expression of at least one of the genes that it was interacting with, with four regions affecting the expression of more than one gene (target gene multiplicity). This validation confirmed the functional importance of the relationships proposed by their pcHi-C maps, but also highlights the importance of using orthogonal methods to demonstrate that the spatial co-localisation of specific elements and genes does lead to downstream consequences on gene expression.

Whalen et al. compared maps of linkage disequilibrium (LD) with maps of chromatin architecture, derived from high-resolution Hi-C, in human and observed that they were largely uncorrelated and reflect the result of two distinct processes (Whalen and Pollard, 2019), with LD resulting from recombination events driven by PRDM9 and chromatin structure resulting from transcription, loop extrusion and other processes (Fudenberg et al., 2016; Rowley et al., 2017; Nuebler et al., 2018). LD blocks were found to be significantly smaller than TADs (median size 13 kb versus 840 kb). Therefore regulatory variants are typically located in LD blocks which do not overlap the gene(s) that they are involved in regulating, with only 2-7% of interactions between genes and noncoding elements being located within the same LD block, highlighting that the range of chromatin interactions is often much larger than the span of LD blocks.

Techniques such as ChIA-PET and HiChIP (Fullwood et al., 2009; Mumbach et al., 2016) combine proximity ligation with an antibody enrichment step, which allows the identification of chromatin interactions associated with a specific transcription factor or histone modification. A study investigating how chromatin interactions differ during the differentiation of embryonic stem cells (ESCs) into neural stem cells (NSCs) and neural progenitors (NPCs) found that the majority of putative enhancers (76%, 77%, 54% for ESC, NSCs and NPCs respectively) did not interact with their nearest gene in any of the cell-types investigated (Zhang et al., 2013). Maps of H3K27ac-associated chromatin interactions generated using HiChIP in distinct cell types from the T-cell lineage found that SNPs associated with autoimmune diseases were enriched in loop anchors. Only 14% of these autoimmune disease-associated SNPs interacted with their nearest gene, with the remainder skipping at least one gene to interact with a more distal gene, a feature the authors termed enhancer skipping (Mumbach et al., 2017).

Large-scale studies of the chromatin interactome have found that regulatory elements often interact with genes that are not linearly proximal, and that these interactions are highly cell-type specific and display enrichment for relevant TFBSs and processes (Beagan and Phillips-Cremins, 2020). These studies help to confirm that disease-associated variants do not need to be in LD to have an effect on the same gene, and that at numerous loci, the range of regulatory influence of an enhancer or SNP is primarily determined by topological organisation, and not by genomic distance. The chromatin interaction landscape is highly complex and cannot be easily predicted from the simple linear genome, due to pervasive features such as enhancer skipping and target gene multiplicity.

## Just how problematic is nearest gene assignment?

**Fig. 4.**
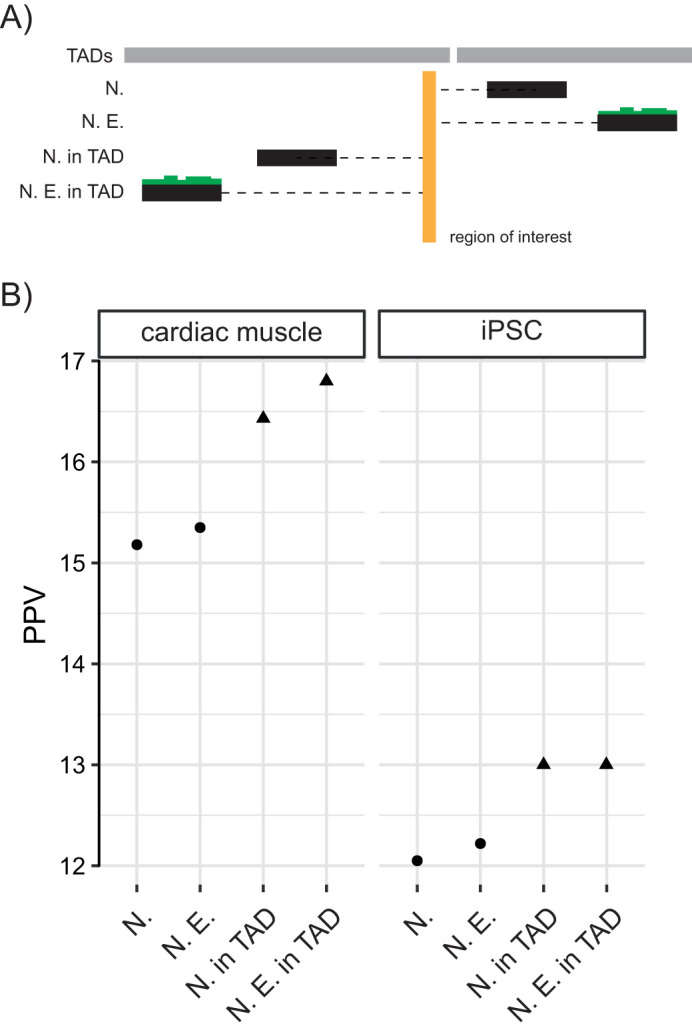
**Performance of nearest gene assignment and associated heuristics in cardiomyocytes.** (A) Schematic of heuristics for assigning a region of interest to its potential target gene **-** nearest gene (N.), nearest expressed gene (N. E.), nearest gene within the same TAD (N. in TAD) and nearest expressed gene within the same TAD (N. E. in TAD). (B) Positive predictive value (PPV) for different heuristics to assign a non-coding region of interest to target genes in cardiac muscle and iPSCs.

Given a non-coding locus of interest there are several potential heuristics for assigning it to its putative target gene(s) (Fig. 4A). A region can simply be assigned the nearest gene in terms of genomic distance (N.) or to the nearest expressed gene (N.E.). N.E. assumes that observing a distal region to be interacting with a gene leads to the gene being expressed. However, given that TADs serve to demarcate the span of interactions between regulatory elements and their targets, incorporating information from Hi-C can be used to reduce the search space and instead ask what the nearest gene within the same TAD (N. in TAD) and nearest expressed gene within the same TAD (N. E. in TAD) are, as the region of interest (Fig. 4A).

By examining genome-wide maps of promoter centered interactions generated using pcHi-C in cardiac muscle (CM) and iPSCs (Montefiori et al., 2018) it is possible to assess the positive predictive value (PPV) for each of these heuristics. PPV (also known as precision) can be interpreted as the probability that an interaction predicted using one of these heuristics is true. For each distal anchor:promoter pair identified using pcHi-C we assessed whether this distal anchor would be assigned to the same gene using each of the four heuristics described above (Fig. 4B).

A PPV of 15.18% and 12.05%, for cardiac muscle (CM) and iPSCs respectively, was observed if only the nearest gene was considered as the target of an anchor. A minor increase in PPV was observed when considering only those genes which are expressed in the relevant cell type. Restricting the search space to only consider genes located within the same TAD as the distal anchor resulted in an increase in PPV to 16.40% and 13.00% for CMs and iPSCs respectively. This highlights that incorporating information on chromatin structure can improve performance in predicting the target of a non-coding region. An increase in PPV was observed only in CM when incorporating information on gene expression. Poor performance on this task was apparent for all of the heuristics investigated. This analysis indicates that studies that use nearest gene assignment (and other related heuristics) lack predictive power for a large number of genes in the genome.

Various algorithms for predicting enhancer–promoter interactions have been developed; for a comprehensive review see (Hariprakash and Ferrari, 2019). These techniques attempt to accomplish this task by using a combination of features including genomic distance, synteny, and one-dimensional (1D) local chromatin states such as transcription factor (TF) binding, histone modifications, and chromatin accessibility signatures.

IM-PET uses a set of four features: correlation between enhancer and target promoter activity profile, transcription factor and target promoter correlation, coevolution of enhancer and target promoter, and a distance constraint between enhancer and target promoter (He et al., 2014). He et al. showed that by incorporating multiple features, IM-PET yielded an area under the curve (AUC) ROC of 94%, which was a significant increase in performance from using only nearest promoter as a predictor. GeneHancer uses combination of genomic distance, eQTL, capture Hi-C, eRNA co-expression, and TF co-expression (Fishilevich et al., 2017). While adding a distance as a feature led to the proposal of ∼500,000 new gene–enhancer connections, the authors noted that none of the ∼40,000 gene–enhancer connections obtained from the most stringent threshold were predicted using distance alone. The activity-by-contact (ABC) model minimally requires a measure of chromatin accessibility in the form of DNase-seq or ATAC-seq data and a measure of enhancer activity, usually H3K27ac (Fulco et al., 2019) and does not consider genomic distance in its predictions, apart from limiting to search space for putative enhancers around potential target genes. Fulco et al. found that predictions based solely on genomic distance achieved an area under the precision-recall curve (AUCPRC) of 0.39, whereas the same metric for the ABC model was 0.65.

In multiple studies attempting to predict enhancer–promoter interactions, genomic distance was found to be a useful feature when considered in combination with other features, but on its own lacks predictive power and has been shown to be a poor predictor of enhancer–promoter interactions.

## Conclusions

The phenomena of enhancers regulating genes other than the one they overlap with or are nearest to is extremely common genome-wide. This has been demonstrated by functional genomics studies dissecting individual gene regulatory domains, as well as from genome-wide studies of enhancer–promoter interactions using chromosome conformation capture techniques. This pattern of long-range regulation is reflected in the conservation of synteny, as observed in comparative genomics studies. All of these studies highlight that for a large number of loci in the genome, nearest gene assignment is wrong. Whilst studies have tried to understand the rules which determine the specificity of enhancer–promoter interactions, we still are lacking a systematic understanding of the features involved (Zabidi et al., 2015; Arnold et al., 2017; Zhou et al., 2020).

When performing genome-wide analysis, it is necessary to consider the impact of the topological organisation of the genome on the robustness of the results and annotation that is being proposed. In attempting to predict the relevant gene given a non-coding locus of interest, not considering information on topological structure and its conservation across both cell types and species and simply assigning a non-coding regulatory element or SNP to the nearest gene will often give misleading results, particularly in the case where the real gene of interest is under long-range regulation. Failure to adequately consider this could lead to incorrect hypotheses about putative causal genes, which would be both time-consuming and expensive. In addition, studies using and developing methods for predicting enhancer–promoter interactions should be first evaluated against nearest gene assignment and other related heuristics to identify whether these machine-learning techniques can first outperform these methods. In addition, it should be remembered that while the prevailing model of enhancer driven regulation is via direct physical interactions, it has been found that some enhancers do not need to be in close physical proximity to regulate gene expression (Benabdallah et al., 2019; Karr et al., 2022).

The development of experimental techniques to assay the regulatory landscape, the development of robust analysis pipelines, and the public availability of high-quality chromosome conformation data will enable researchers to drastically reduce the search space of potential target genes, which when followed by further computational analysis and experimental validation will help improve our mechanistic understanding of gene regulation and how its dysregulation impacts disease.

## MATERIALS AND METHODS

Validated *Shh* enhancers were obtained from Jeong et al., Sagai et al. and Tsukiji et al. and lifted over to mm10 using rtracklayer (Jeong et al., 2006; Sagai et al., 2009; Tsukiji et al., 2014)*.* Sets of conserved non-coding elements (CNEs) were obtained from ANCORA and smoothed using a sliding window approach to generate density tracks (Engström et al., 2008).

4C-seq from the brain of embryonic (E14.5) and adult mice was obtained from Smemo et al. and lifted over from mm9 to mm10 using rtracklayer (Smemo et al., 2014).

RNA-seq data for iPSC and cardiac muscle was obtained from E-MTAB-6013 (Montefiori et al., 2018) and aligned against the human genome (hg19 Ensembl 87) using STAR and quantified using RSEM (Dobin et al., 2013; Li and Dewey, 2011).

mESC Hi-C data was obtained from GEO:GSE96107 (Bonev et al., 2017) and aligned using BWA against mm10 and processed using FAN-C (Kruse et al., 2020). Aligned data was filtered and binned into 40 kb bins and KR-normalised. TADs were identified using TopDom (Shin et al., 2016). Hi-C data for iPSC and cardiac muscle was downloaded from E-MTAB-6014 (Montefiori et al., 2018), aligned using BWA and processed using FAN-C. Aligned data was filtered and binned into 40 kb bins and KR-normalised. TADs were identified using TopDom. Promoter capture Hi-C data for iPSC and cardiac muscle was obtained from Montefiori et al. (2018), and was analysed using GenomicInteractions (Harmston et al., 2015). Visualisations of genomic data were generated using a combination of GViz (Hahne and Ivanek, 2016) and GenomicInteractions (Harmston et al., 2015).

For calculating the positive predictive value of various heuristics, interactions spanning longer than 2Mb were removed from the pcHi-C datasets. Genes which were expressed at more than 1 TPM in at least one replicate of cardiac muscle cells or iPSCs were defined to be expressed in that cell type. A true positive was defined if the gene predicted by a heuristic matched one of the genes that that the anchor/bait region was found to be interacting with (as in some cases a bait region can overlap the promoters of different genes). For nearest gene (N.) we identified which was the closest gene in terms of genomic distance to each of the anchors of interest, whereas for nearest expressed gene (N.E.), we first filtered out all genes that were not expressed at more than 1 TPM in at least one replicate in the corresponding cell line, we next identified which of these genes were closest to each of the anchors of interest. For assessing the performance of using the nearest gene in the same TAD (N. in TAD), we used TADs identified using TopDom to restrict the search space for potential genes and only calculated distances between genes and anchors of interest for genes which were present in the same TAD. For nearest expressed gene within the same TAD (N.E. in TAD), only those genes with a TAD that were expressed at more than one TPM In at least one replicate were considered. Results obtained from including Hi-C data (TADs) were robust to the choice of the parameter *w*.

All code necessary to recreate figures and analyses from this manuscript are available from: https://github.com/harmstonlab/NearestGene
